# Supplementary material for: Proenkephalin as a Novel Prognostic Marker in Heart Failure Patients: A Systematic Review and Meta-Analysis
Source: Int J Mol Sci. 2023 Mar 3;24(5):4887. doi: 10.3390/ijms24054887 (PMC10003589; doi:10.3390/ijms24054887)
Supplement: Supplementary file 1 [file ijms-24-04887-s001.zip › ijms-2149101-supplementary.pdf]

Table S1. Search terms for systematic review.

Databases: Ovid MEDLINE, EMBASE, and Cochrane Database

1. exp heart failure/
2. heart failure$.mp
3. 1 or 2
4. exp leu-enkephalin /
5. leu-enkephalin$.mp
6. exp met-enkephalin /
7. met-enkephalin$.mp
8. exp proenkephalin /
9. proenkephalin$.mp
10. exp enkephalin /
11. enkephalin$.mp
12. 4 or 5 or 6 or 7 or 8 or 9 or 10 or 11
13. 3 and 12
